# A framework of computer vision-enhanced microfluidic approach for automated assessment of the transient sickling kinetics in sickle red blood cells

**DOI:** 10.3389/fphy.2024.1331047

**Published:** 2024-03-14

**Authors:** Yuhao Qiang, Mengjia Xu, Mira Patel Pochron, Madhulika Jupelli, Ming Dao

**Affiliations:** 1 Department of Materials Science and Engineering, Massachusetts Institute of Technology, Cambridge, MA, United States,; 2 Department of Data Science, Ying Wu College of Computing, New Jersey Institute of Technology, Newark, NJ, United States,; 3 Center for Brains, Minds and Machines, Massachusetts Institute of Technology, Cambridge, MA, United States,; 4 Pfizer Inc., New York, NY, United States

**Keywords:** deep learning, microfluidics, image segmentation and classification, drug testing, sickle cell disease, automated sickling kinetics assay

## Abstract

The occurrence of vaso-occlusive crisis greatly depends on the competition between the sickling delay time and the transit time of individual sickle cells, i.e., red blood cells (RBCs) from sickle cell disease (SCD) patients, while they are traversing the circulatory system. Many drugs for treating SCD work by inhibiting the polymerization of sickle hemoglobin (HbS), effectively delaying the sickling process in sickle cells (SS RBCs). Most previous studies on screening anti-sickling drugs, such as voxelotor, rely on *in vitro* testing of sickling characteristics, often conducted under prolonged deoxygenation for up to 1 hour. However, since the microcirculation of RBCs typically takes less than 1 minute, the results of these studies may be less accurate and less relevant for in vitro-in vivo correlation. In our current study, we introduce a computer vision-enhanced microfluidic framework designed to automatically capture the transient sickling kinetics of SS RBCs within a 1-min timeframe. Our study has successfully detected differences in the transient sickling kinetics between vehicle control and voxelotor-treated SS RBCs. This approach has the potential for broader applications in screening anti-sickling therapies.

## Introduction

**FIGURE 1:**
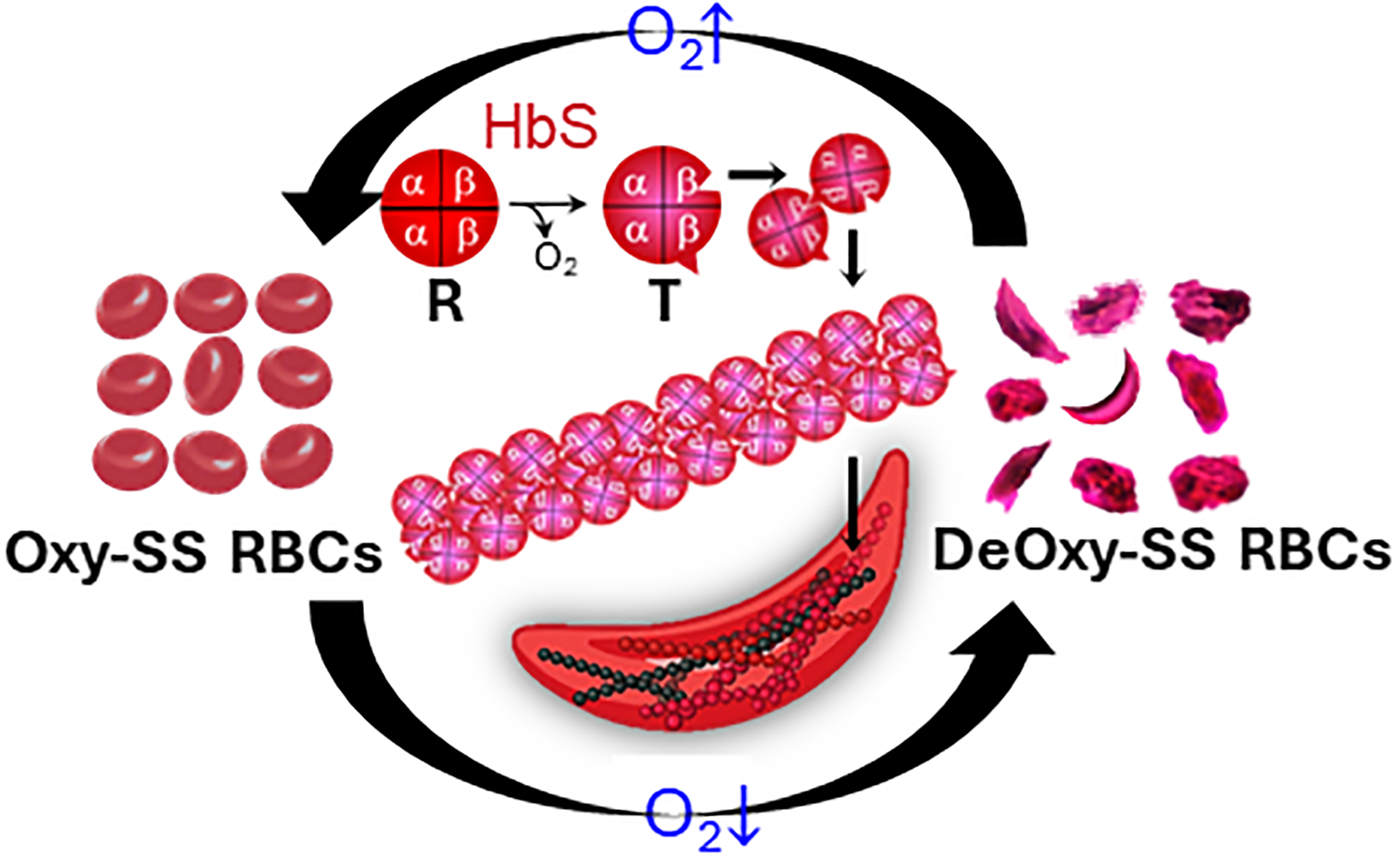
Deoxygenation leads to sickling and morphological changes of sickle RBCs. The exposure of β6 valine and its binding with complementary hydrophobic site on β-hemoglobin induces the polymerization and fiber formation of Hb S while switching from R conformation to T conformation.

Sickle cell disease (SCD) is a hereditary hematologic disorder resulting from a mutation in the β subunits of hemoglobin within red blood cells (RBCs) [1, 2]. Upon deoxygenation, the sickle hemoglobin (HbS) within sickle cells (SS RBCs) is subject to the conformational change from the relaxed (R) state to the tense (T) state. The exposure of β6 valine and its binding with the complementary hydrophobic site on β-hemoglobin induces the transient polymerization and long fiber formation of intracellular HbS, accompanied by membrane crenation and stiffening of RBCs (Figure 1) [3, 4]. This sickling process predominantly contributes to the complications of SCD, including obstruction in the microvasculature such as postcapillary venules and splenic inter-endothelial slits (IESs) [5–7], hemolytic anemia, life-threatening organ damage and even death [7–9].

Prior studies have evolved to the point that the competition between transient sickling delay time and transit time of individual RBCs passing through micro-constrictions *in vivo* is crucial in determining the occurrence of vaso-occlusive crisis (VOC) in SCD patients [10–12]. Some earlier studies have indicated that the treatment of anti-sickling compounds, such as voxelotor, can effectively slow down the sickling process and decrease the proportion of sickled RBCs [13–15]. However, these studies have a limitation in that they are conducted under conditions of prolonged deoxygenation (>1 h), which is two orders of magnitude longer than the duration of the systemic blood circulation (~30s) [13, 16] and even the sluggish splenic circulation (~1min) [7, 17]. Interpreting the results of these studies as indicative of the impact of drug treatment on the transient-state sickling kinetics of individual SS RBCs and its potential to reduce the likelihood of VOC in SCD becomes challenging due to the significant disparity in the experimental conditions compared to the physiological context. The extended deoxygenation periods used in these studies may not accurately reflect the transient-state sickling kinetics occurring during the rapid circulation of blood, making it difficult to draw direct conclusions regarding the efficacy of drug treatment in preventing VOC in SCD patients [13]. More recently, sickling kinetics assays under monotonic and cyclic transient hypoxia have been successfully developed [18–20], where the transient sickling behavior of SS RBCs within 1–2 min can be accurately quantified. However, the quantification of sickling kinetics in these studies was conducted manually, which is extremely time-consuming and very inefficient. There is a pressing and unmet need for a more efficient and effective method to capture the transient sickling behavior of SS RBCs. This need is especially crucial when screening anti-sickling drugs. Developing more relevant and efficient approaches for studying these dynamics is essential for advancing research and treatment options for SCD.

In this paper, we introduce a novel framework that combines computer vision with microfluidic approaches to enable rapid assessment of the sickling kinetics in SS RBCs under transient deoxygenation. The sickling kinetics can be quantified by the time courses of transient shape factors of each RBC, as well as the overall sickled fraction across a cell population as they undergo the sickling process. We have also utilized our approach to conduct a pilot study evaluating the effectiveness of the anti-sickling drug, voxelotor, on SS RBCs. We envision this method could be generally applicable to the drug testing of anti-sickling therapies.

## Methods

### Sample preparation

**FIGURE 2:**
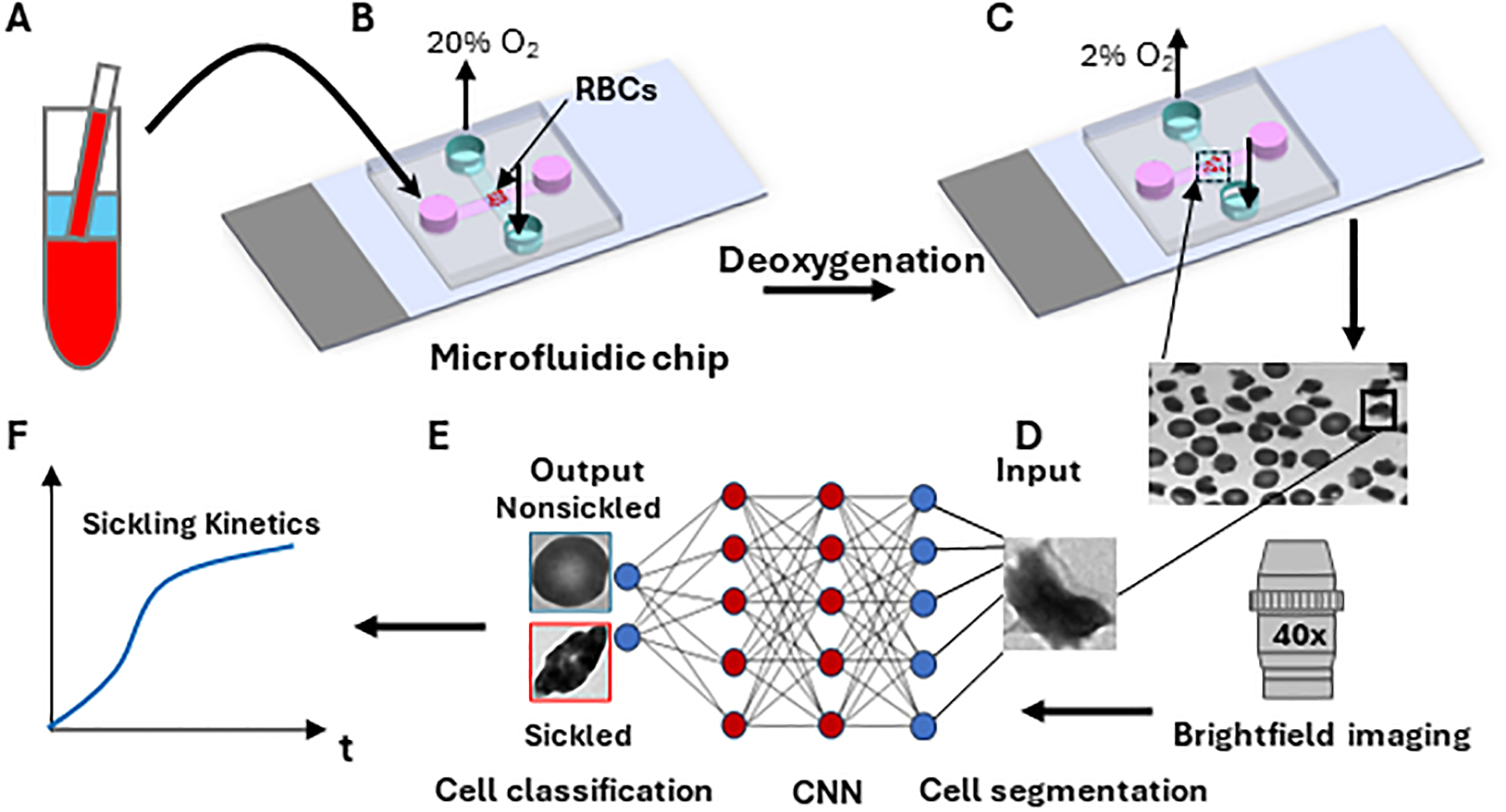
Overall experimental approach for sickling kinetics testing. **(A)** RBC sample preparation. **(B)** RBCs are transferred to the microfluidic device. **(C)** Transient sickling of RBCs through rapid on-chip deoxygenation. **(D)** Microscopic cell imaging using a ×40 objective. **(E)** Structure of the deep convolutional neural network (CNN) for image-based cell classification. **(F)** Automated measuring of sickling kinetics of SS RBCs.

Sickle blood samples were obtained from individuals with homozygous sickle cell patients at the University of Pittsburgh, following the guidelines and ethical standards established in the Institutional Review Board (IRB) protocol PRO08110422. The normal blood sample was drawn from a healthy subject at the Massachusetts General Hospital under an Excess Human Material Protocol approved by the Partners Healthcare Institutional Review Board (IRB) with a waiver of consent. Blood samples were then promptly transported to MIT for experiments under an approved exempt protocol (Massachusetts Institute of Technology IRB protocol E−1523). Upon measurements, blood samples were washed twice with phosphate-buffered saline (PBS, Lonza Walkersville, Inc., Walkersville, MD) at 2000 rpm for 2 min at room temperature. RBC suspensions were adjusted to 20% Hct and incubated with voxelotor (Pfizer Inc., South San Francisco, CA) targeting 30% modification of RBC hemoglobin or dimethyl sulfoxide (vehicle) in PBS containing 1% (w/v) bovine serum albumin (BSA) (EMD Millipore, Billerica, MA) for 60 min in the 37 °C water bath. After treatment, RBCs were washed twice with PBS at 2000 rpm for 2 min at room temperature, and then diluted to 10^6^ cells/mL in the PBS with 1% (w/v) BSA before injection into the microfluidic device for sickling kinetics testing (Figure 2A).

### Sickling kinetics testing assay

The microfluidic devices used for the sickling kinetics testing were fabricated following standard fabrication processes [18–20]. The microfluidic devices were composed of two microchannels, which were aligned perpendicular to each other and separated by an oxygen-exchange porous polydimethylsiloxane membrane (150 μm thick) in the cross-sectional area. The transient deoxygenation condition was achieved by switching the gas supply in the upper channel from a high oxygen level (20% oxygen (O_2_), 5% carbon dioxide (CO_2_) with the balance of nitrogen (N_2_)) for 30 s to a low oxygen level (2% O_2_, 5% CO_2_ with the balance of N_2_) for 120 s (Figures 2B, C). Both the upper gas channel and the lower cell channel are 1,500 μm wide and 150 μm deep. The gas supply pressure was regulated to be ~3.5 psi, and the time taken for oxygen to traverse the 150-micron thick membrane was measured to be around 10s [20]. The maximum deflection at the center of the 150-micron thick membrane in the gas channel was estimated to be ~0.5 um based on Roark’s formulas for stress and strain (for flat plates) [21], suggesting that the gas pressure barely affects the structural integrity of the microfluidic channels and RBCs. The sickling kinetics testing was performed in a hydrostatic condition right after the suspended RBCs sedimented to the substrate in the microchannel. The sickling processes of SS RBCs were imaged and recorded through a high-resolution CMOS camera (The Imaging Source, Charlotte, NC, USA) which was mounted on a Zeiss Axiovert 200 inverted microscope (Carl Zeiss Inc., Thornwood, NY) with a ×40 objective lens (Figure 2D).

### Automated image analysis

The image data was extracted from the recorded microscopic videos in the form of a series of 16-bit grayscale, 720 × 480-pixel images, and saved as PNG files for subsequent analysis (Figures 2E, F). For the single cell contour analysis, we computed multiple shape factors, including Circular shape factor (*CSF* = 4 *π* ×*Area_r*/*Perm_r*^2^), Ellipticity shape factor (*ESF* = *Rb*/*Ra*), *SF1*(=*Rb*/*max_FD*), *SF2* (=*min_FD*/*max_FD*), *Elongation* (=*max_FD*/*min_FD*), $\text{Convexity }\left(=\;\sqrt{\text{Area}\_r/\text{Area}\_c}\right)$ and *Compactness* (=4 *π* ×*Area_r*/*Perm_c*^2^), where *Rb and Ra are the* minor axis and major axis of the fitted ellipse of the cell*, Area_r* and *Perm_r* are the segmented area and perimeter of the cell, *max_FD* and *min_FD* are the maximum Feret diameter and minimum Feret diameter, and *Area_c* and *Perm_c* are the convex area and convex perimeter, respectively [22]. Skewness and Kurtosis were computed for the intensity distribution analysis. The image contour and intensity analysis were conducted using a custom script in Matlab R2022a (MathWorks, Natick, MA). The algorithms of single cell segmentation and classification using deep convolutional neural networks (CNNs) were implemented in Jupyter Notebook based on Python open-source libraries including Cellpose, Numpy, Tensor Flow, and Matplotlib, *etc*. All runs were performed on a PC with an NVIDIA GeForce RTX 3070 GPU. The architecture of our CNN consists of 17 layers, including 5 convolutional layers (C1, C3, C6, C9, and C12), 5 pooling layers (P2, P4, P7, P10 and P13), 5 dropout layers (D5, D8, D11, D14 and D17, with probability of 0.001), a flatten layer (F15) and a fully connected layer (FC16). All convolutional layers had several kernels each with the size of (3, 3). The (2, 2) max-pooling layers were implemented after the convolutional layers for downsampling the feature maps. For the activation function, the ReLU non-linear function was used. A softmax function with a categorical cross-entropy loss function was applied for the final training and prediction with two outputs (nonsickled and sickled). The performance of our classification model was evaluated using several performance metrics including *Accuracy* (= (*TP* + *TN*)/(*TP* + *TN* + *FP* + *FN*)), *Precision* (= *TP*/(*TP* + *FP*)), *Recall* (= *TP*/(*TP* + *FN*)), *F1-score* (= 2 × (*Precision* × *Recall*)/(*Precision* + *Recall*)), where *TP*, *TN*, *FP*, and *FN* represent True Positive, True Negative, False Positive, and False Negative, respectively.

## Results

### Cellular segmentation in time-lapse image sequences during the sickling process

**FIGURE 3:**
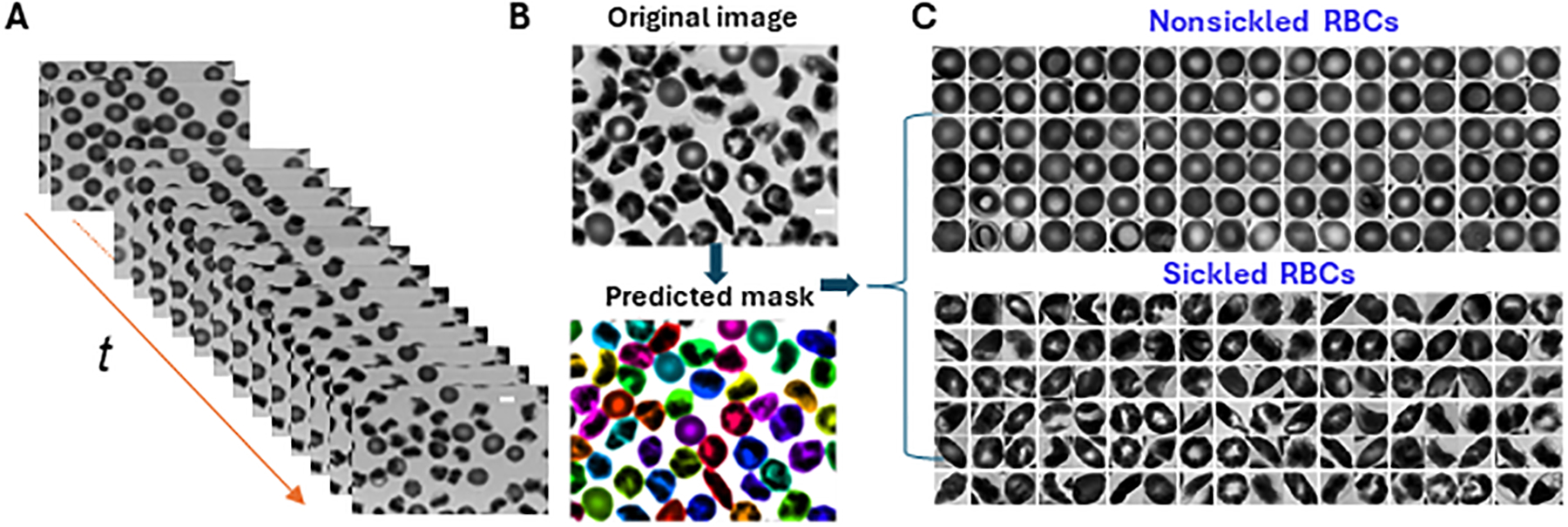
Cellular segmentation in time-lapse image sequences. **(A)** The stack of microscopic image sequences of the time course during cellular sickling. **(B)** An instance of cellular segmentation using the Cellpose algorithm. Original image (top) and predicted masks (bottom). **(C)** Image annotation by labeling segmented image patches into two subsets of nonsickled RBCs and sickled RBCs, respectively. (Scale bars: 5 μm).

To analyze the dynamics of cellular behavior over time during the sickling process, we used a Cellpose algorithm [23, 24] to automatically segment the image sequences exported from the recorded microscopic videos (Figure 3A). The segmentation model was retrained on a small set of 50 randomly selected images based on the pre-trained cytoplasm model in Cellpose using its interactive annotation and model retraining platform (Figure 3B). The average cell diameter was set to be 90 pixels, which could enable us to exclude the small amount (<1%) of overlapping cells. The image was automatically cropped to obtain single RBC images. During the identification process, the cells majorly located outside the frame of the image were discarded. In preparation for the subsequent classification training process, the segmented individual cell patches were further processed by annotating or labeling the cellular images. They were categorized into two distinct subsets: nonsickled RBCs and sickled RBCs, respectively. (Figure 3C). The annotation process was accomplished by a human annotator through visual identification based on the distinct morphological and texture differences, i.e., sickled RBCs exhibit peculiar shapes and dark coarse texture, whereas the nonsickled RBCs present regular shapes and light smooth texture [18]. To note, we considered those irreversibly sickled cells (ISCs) in the initial condition of the sickling process as “sickled” RBCs in this study.

### Cellular shape factor extraction of individual nonsickled and sickled RBCs

**FIGURE 4:**
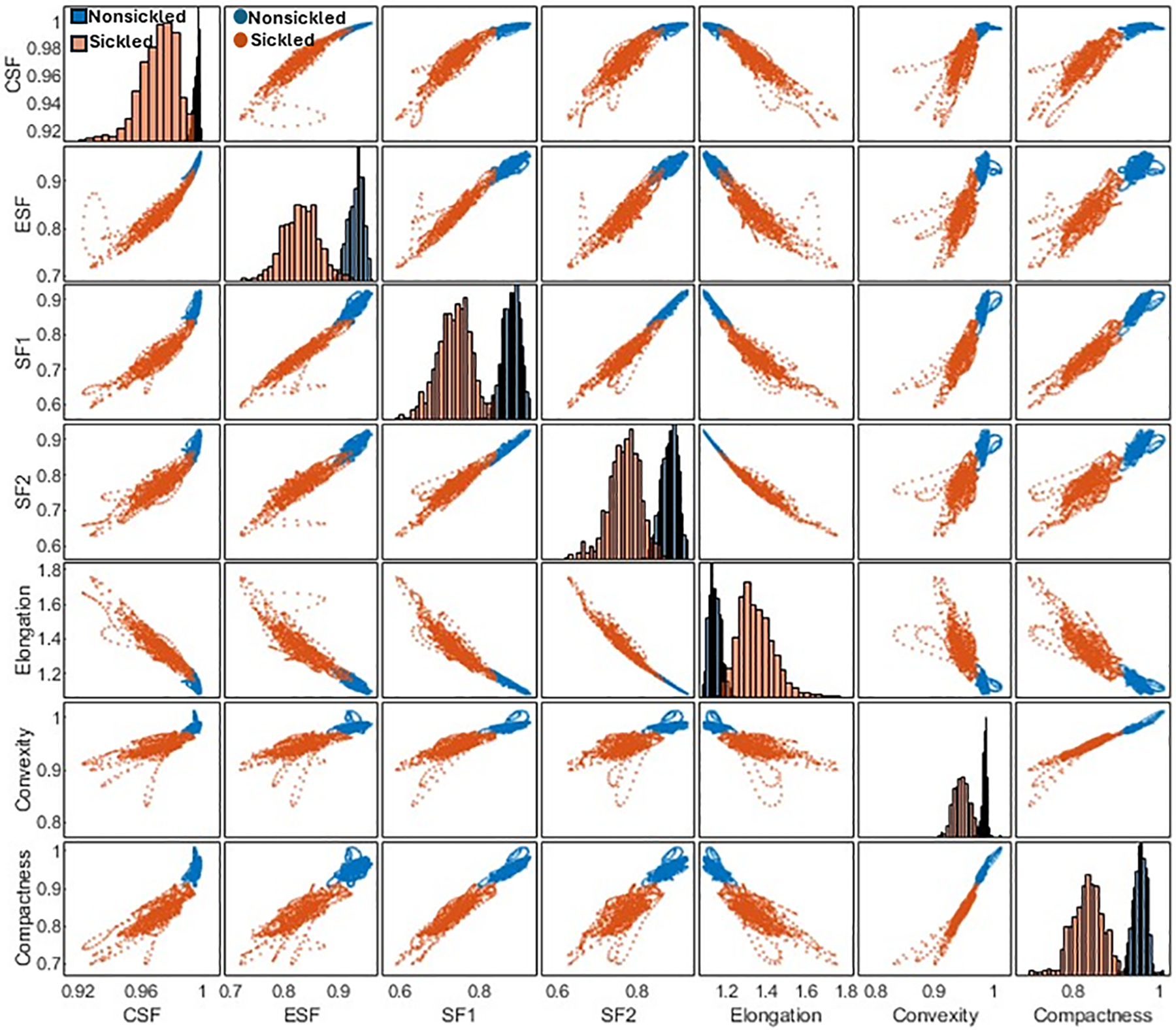
Correlation matrix plot for cellular shape factors, including the *CSF*, *ESF*, *SF1*, *SF2*, *Elongation*, *Convexity*, and *Compactness* of single SS RBCs annotated in two subsets of nonsickled and sickled RBCs, respectively.

Following the segmentation and annotation processes, the study proceeded with a detailed analysis of the shape factors for each RBC within both cell types. Seven shape factors, including the *CSF*, *ESF*, *SF1*, *SF2*, *Elongation*, *Convexity*, and *Compactness*, were automatically computed through the contour analysis. See Methods for the detailed computational formulas of the RBC shape factors. Figure 4 shows the matrix plot of the seven shape factors for 1,560 nonsickled cells and 1,270 sickled RBCs, respectively. The 2 cell types have shown distinct clusters in the scatterplots of the shape factors. In contrast to the nonsickled RBCs, those sickled RBCs cover a broader range of distribution, suggesting a higher heterogeneity of morphologies in sickled RBCs. For example, the plots of the nonsickled RBCs are superimposed at the right top corner of the *CSF*-*ESF* scatterplot, while the plots of the sickled RBCs are much more widely scattered and spread down to the left bottom corner. The results indicate that the nonsickled RBCs and sickled RBCs exhibit distinct features in morphology, which can be readily used to classify and differentiate between these 2 cell types.

### Temporal evolution of the sickling profiles of single SS RBCs

**FIGURE 5:**
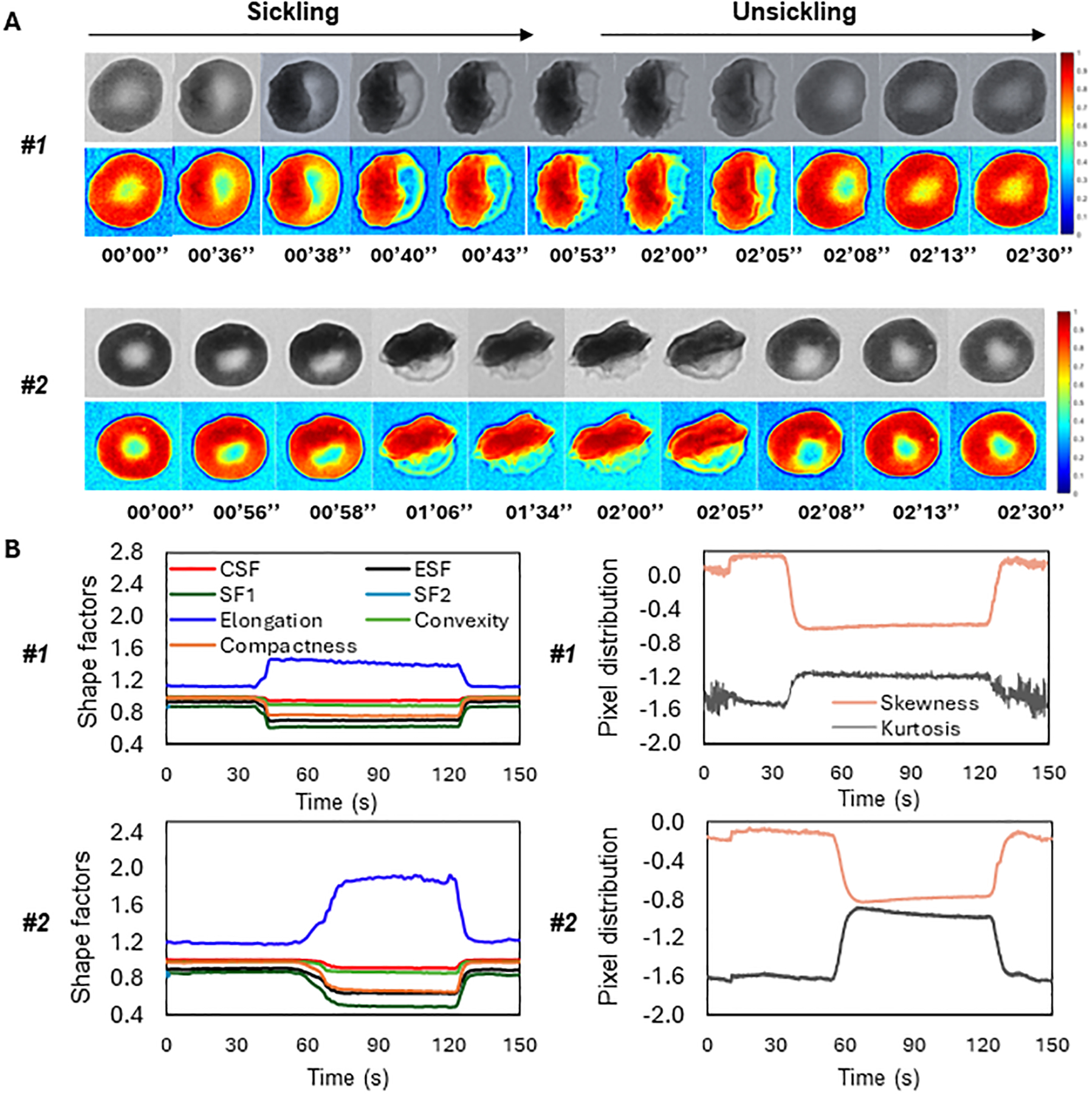
Temporal evolution of shape factors in single SS RBCs through automated image analysis. **(A)** The microscopic image sequences of two representative SS RBCs (Cell #1 and Cell #2), showing the distinct differences in sickling and unsickling rate. Original image (top) and colormap (bottom). (Scale bar: 5 μm) **(B)** The corresponding profiles of shape factors (left) and pixel distributions (right) of the two representative SS RBCs as a function of time.

Figure 5A shows the time-lapse microscopic images of two representative SS RBCs (Cell #1 and Cell #2) during the sickling and unsickling processes, respectively. It appears that significant changes in both the shape and texture of SS RBCs were clearly observable during both the sickling and unsickling processes. In comparison, these two representative SS RBCs show distinct dynamic sickling processes in response to the same deoxygenation condition. Cell #1 underwent rapid sickling, completing the process within less than 36 s, whereas Cell #2 began the sickling process after 56 s. In contrast, we noticed that the process of unsickling, or returning to a normal state, occurred much more rapidly compared to the initial sickling process of the same SS RBC. Notably, there was no significant difference observed in the unsickling process between these two RBCs. In Figure 5B, the sickling profiles of various shape factors, including *CSF*, *ESF*, *SF1*, *SF2*, *Elongation*, *Convexity* and *Compactness* and intensity distribution factors (*Skewness* and *Kurtosis*) are displayed as a function of time, which are generated through the automated contour and intensity analyses. All the curves demonstrate transient shape changes, in particular in terms of the *Elongation, SF1, SF2, ESF, and Compactness*, during both the sickling and unsickling, indicating that the sickling kinetics in single SS RBCs can be effectively tracked using the parameters derived from automated cellular image analysis. The changes in these parameters over time provide valuable information about the dynamic behavior of RBCs as they transition between their normal and sickled states, contributing to a better understanding of the sickling process in single SS RBCs.

### Automated classification of SS RBC sickling yields through deep-learning-enabled image analysis

**FIGURE 6:**
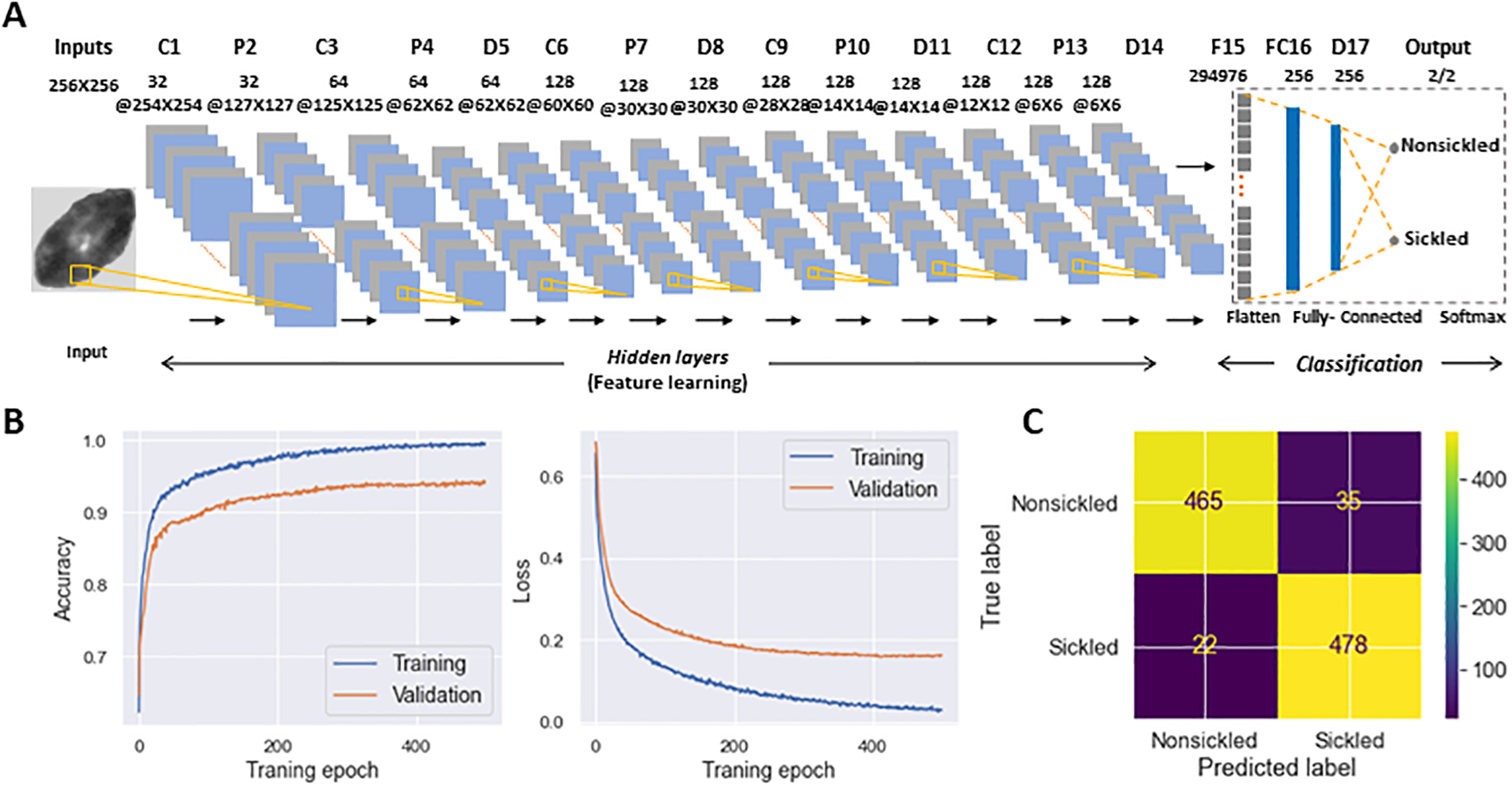
Classification of SS RBC sickling status through deep-learning-enabled cellular image analysis. **(A)** Diagram detailing the architecture of deep convolutional neural network used for SS RBC classification. **(B)** Training and validation history of the accuracy (left) and loss function (right) over 500 epochs. **(C)** Confusion matrix for the classification of nonsickled RBCs and sickled RBCs. The intensity bar represents the scale.

To evaluate the temporal sickling profile within a population of cells, we designed a deep CNN model for the automated classification of individual SS RBCs into two categories, i.e., sickled and nonsickled RBCs, in each frame of the raw image. The details of the CNN architecture are shown in Figure 6A and are further described in Methods. A total of 1993 nonsickled RBC images and 2011 sickled RBC images were used for the training of the CNN model. We split the training dataset into two parts: 80% was used for training (training set), and the remaining 20% was allocated for validation (validation set). All input images were normalized to the same dimensions of 256 × 256 pixels for training. We further augmented the training set by applying random transformations to each image after each epoch, including a random shifting by 10% of the total width and random flips in both horizontal and vertical directions. We used the Adam optimizer for the model optimization. We evaluated our model with various learning rates and numbers of epochs, ultimately selecting the best to reduce overfitting and training loss. As a result, the optimal model was trained at the learning rate of 0.001 for 500 epochs. Figure 6B shows the performance of training and validation in terms of classification accuracy and log-loss over 500 epochs. Figure 6C shows the corresponding confusion matrix for the validation set. The validation *Accuracy*, *Precision*, *Recall*, and *F1-score* of the trained network were all evaluated to be 0.94, respectively.

**FIGURE 7:**
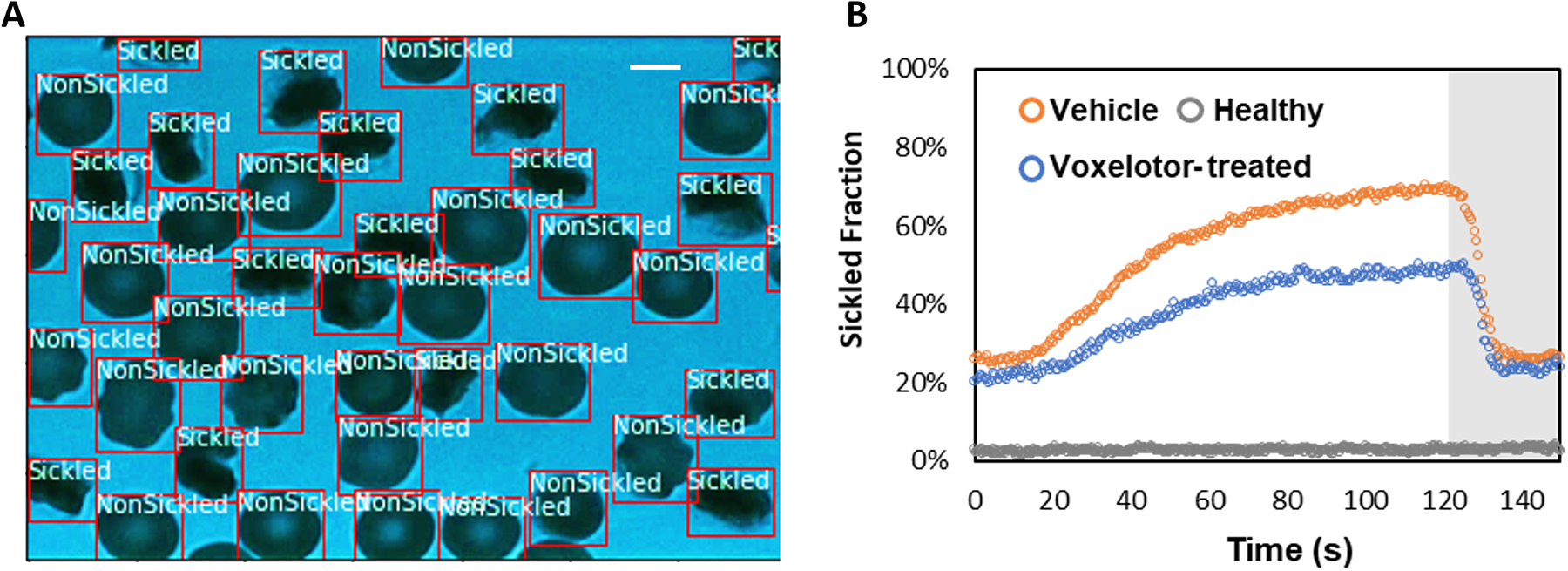
Temporal evolution of SS RBC sickling yields through the deep CNN prediction. **(A)** Real-time classification of sickling across a cell population. The two classes (nonsickled and sickled) in the RBCs are detected and localized with bounding boxes. (Scale bar: 5 μm) **(B)** Profiles of sickled fraction in normal RBCs (n = 212), vehicle control SS RBCs (n = 291) and voxelotor-treated SS RBCs (n = 191) during 120s of sickling (white region) followed by 30s of unsickling process (gray region), measured through automated image classification.

To evaluate the temporally evolving sickling kinetics of SS RBCs across a cell population, the trained CNN model was applied to classify all the RBCs frame-by-frame throughout the image sequences (Figure 7A). The time course of sickling yields was then automatically computed by the real-time fraction of the sickled RBCs. As shown in Figure 7B, we utilized this classification scheme to test the efficacy of voxelotor in a pilot study, where we could sensitively detect the difference in the transient sickling kinetics between the vehicle control and voxelotor-treated SS RBCs from the same SCD patient blood sample following a successive deoxygenation and reoxygenation processes. Our results have also shown that there is about 20% of RBCs differentiated as “sickled” RBCs in the initial oxygenation state, which is due to the preexistence of a small fraction of ISCs [25, 26].

## Discussion and concluding remarks

In the present study, a framework has been established that combines computer vision and microfluidic techniques to autonomously evaluate the transient sickling kinetics of SS RBCs, which is crucial in determining the probability of VOC occurrence in SCD. The incorporation of computer vision techniques allows for the automated segmentation and recognition of sickled RBCs. The utilization of computer vision techniques offers a substantial enhancement in the efficiency and accuracy of data analysis when compared to the human identification method previously employed in our prior work [18–20]. This improvement is particularly notable in the context of cyclic sickling evolution studies, where automation not only speeds up the analysis process but also provides more consistent and objective results. It reduces the potential for human error and subjectivity, thereby advancing the quality and reliability of the research. The improved assessment of the transient sickling behaviors in single RBCs can also improve the accuracy in the mesoscale modeling and simulations [27–29]. From the analysis of raw images, we can see SS RBCs exhibit notable morphological changes in multiple geometric and textural features after sickling, which could give us *a priori* explanation for the CNN to identify the sickling process. This can also be clearly seen from obvious changes in several shape factors shown in Figure 4. We have further concatenated the shape factors (the *CSF*, *ESF*, *Convexity*, and *Compactness*) for the data fusion with the input images in training our CNN, and obtained the same performance as shown in Figure 6B (*Accuracy* = 0.94, *Precision* = 0.94, *Recall* = 0.94, *F1-score* = 0.94). This exercise suggests that the CNN shown in Figure 6 can capture various shape changes in sickled RBCs well. Many aspects can be further improved in our forthcoming work. One such aspect is our current differentiation of SS RBCs into only two categories: “nonsickled” cells and “sickled” cells. Future work could be done to explore more details of the evolution of various shape subcategories within “sickled” as well as “nonsickled” RBCs throughout the sickling process. Another interesting aspect is to explore further the cell sickling process in terms of “explainable artificial intelligence”.

Under physiological conditions, microcirculation and the occurrence of sickling events in SS RBCs typically unfold on a timescale of merely seconds. Prior investigations have unveiled that the administration of anti-sickling agents, such as voxelotor, can substantially diminish the proportion of sickled RBCs after a prolonged period of deoxygenation [13–15]. Nonetheless, our previous study has demonstrated that even a minor fraction of impassable RBCs can result in severe blockages within the micro-constrictions of small capillaries [7]. Furthermore, earlier research has demonstrated that the morphology of sickled RBCs is markedly influenced by the rate of deoxygenation [30, 31]. Therefore, to a certain extent, the short-term transient sickling kinetics of individual SS RBCs hold greater significance than the proportion of sickled cells following a prolonged period of deoxygenation. Crucially, it is imperative to assess the effectiveness of anti-sickling drugs on the sickling rate of individual SS RBCs during transient deoxygenation, a capability that our computer vision-enhanced approach uniquely offers. We anticipate that this approach could find broad applicability for high-throughput and rapid screening of various compounds aimed at anti-sickling therapies.

## Data Availability

The raw data supporting the conclusion of this article will be made available by the authors, without undue reservation.
